# Distribution of circular proteins in plants: large-scale mapping of cyclotides in the Violaceae

**DOI:** 10.3389/fpls.2015.00855

**Published:** 2015-10-27

**Authors:** Robert Burman, Mariamawit Y. Yeshak, Sonny Larsson, David J. Craik, K. Johan Rosengren, Ulf Göransson

**Affiliations:** ^1^Division of Pharmacognosy, Department of Medicinal Chemistry, Uppsala UniversityUppsala, Sweden; ^2^Department of Pharmacognosy, School of Pharmacy, Addis Ababa UniversityAddis Ababa, Ethiopia; ^3^Craik Lab, Chemistry and Structural Biology Division, Institute for Molecular Bioscience, The University of QueenslandBrisbane, QLD, Australia; ^4^Laboratory for Peptide Structural Biology, School of Biomedical Sciences, The University of QueenslandBrisbane, QLD, Australia

**Keywords:** cyclotide, Violaceae, plant protein, plant peptide, stable proteins

## Abstract

During the last decade there has been increasing interest in small circular proteins found in plants of the violet family (Violaceae). These so-called cyclotides consist of a circular chain of approximately 30 amino acids, including six cysteines forming three disulfide bonds, arranged in a cyclic cystine knot (CCK) motif. In this study we map the occurrence and distribution of cyclotides throughout the Violaceae. Plant material was obtained from herbarium sheets containing samples up to 200 years of age. Even the oldest specimens contained cyclotides in the preserved leaves, with no degradation products observable, confirming their place as one of the most stable proteins in nature. Over 200 samples covering 17 of the 23–31 genera in Violaceae were analyzed, and cyclotides were positively identified in 150 species. Each species contained a unique set of between one and 25 cyclotides, with many exclusive to individual plant species. We estimate the number of different cyclotides in the Violaceae to be 5000–25,000, and propose that cyclotides are ubiquitous among all Violaceae species. Twelve new cyclotides from six phylogenetically dispersed genera were sequenced. Furthermore, the first glycosylated derivatives of cyclotides were identified and characterized, further increasing the diversity and complexity of this unique protein family.

## Introduction

During recent decades plants have increasingly been recognized as an important source of bioactive proteins. As well as being attractive systems for protein production (Yusibov et al., 2011; Sack et al., 2015), plants provide unique protein scaffolds that are suitable for applications in drug design and agriculture (Boothe et al., 2010; Craik et al., 2010; Padovan et al., 2010; Göransson et al., 2012). Of particular interest from this applied perspective are small cysteine-rich proteins, which are highly stable and adaptable to provide many biological activities. One such family of plant proteins that possesses unique properties is the cyclotides (cyclo-peptides) that are found among species of violets (Violaceae). Cyclotides comprise a circular chain of approximately 30 amino acids, including six conserved cysteines that form the three disulfide bonds creating the cyclic cystine knot (CCK) motif (Craik et al., 1999). This motif imparts cyclotides with exceptional thermal, enzymatic, and chemical stability (Colgrave and Craik, 2004). Apart from the conserved cysteines, the sequences of cyclotides are highly diverse, suggesting that the rigid scaffold can be adopted to accommodate a wide range of biological activities, making them ideal candidates for protein engineering ventures (Chan et al., 2011; Aboye et al., 2012; Wong et al., 2012; Koehbach et al., 2013b; Wang et al., 2014).

**Figure 1:**
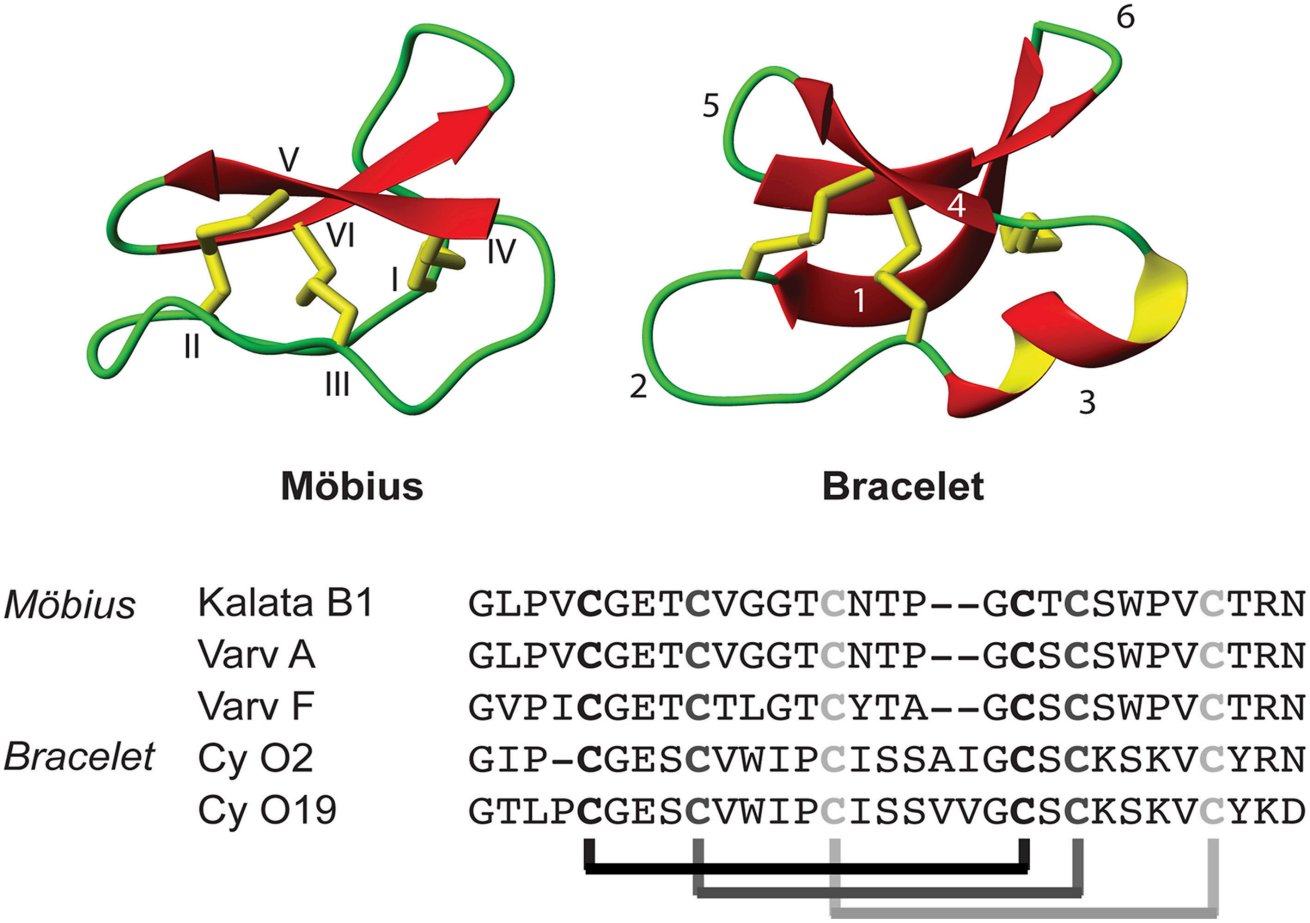
**Schematic structure of a Möbius and bracelet cyclotide, together with typical cyclotide sequences from both subfamilies**. The structures are based on the PDB files 1NB1 and 2KNM. The abbreviated notation Cy O2 and Cy O19 stand for the cycloviolacins O2 and O19, respectively. Note the unique features of the CCK motif: a cyclic backbone with sequence loops (1–6) and three stabilizing disulfide bonds. These disulfides are arranged in a cystine knot: that is, two of the disulfides form a ring structure together with the backbone connecting the four cysteines (I–IV; II–V), while the third disulfide is threaded through the ring (III–VI).

A number of biological activities have already been described for naturally occurring cyclotides, including cytotoxic effects (Lindholm et al., 2002; Svangård et al., 2004; Herrmann et al., 2008), hemolytic (Daly and Craik, 2000), anti-HIV (Gustafson et al., 2004; Wang et al., 2008a), and anti-fouling properties (Göransson et al., 2004). They have also been shown to have insecticidal (Jennings et al., 2001, 2005; Barbeta et al., 2008), nematocidal (Colgrave et al., 2008), and antimicrobial (Tam et al., 1999; Pränting et al., 2010; Ovesen et al., 2011) properties, suggesting that their biological function is probably linked to host defense of plants. Recent studies have shown that cyclotides interact with cell membranes (Svangård et al., 2007; Huang et al., 2009; Burman et al., 2011; Henriques and Craik, 2012), a phenomenon consistent with their broad range of biological effects. To date, more than 300 cyclotides have been described and they are generally divided into two main subfamilies, namely the Möbius and the bracelet subfamilies, which are characterized by the presence or absence, respectively, of a conserved *cis* proline (Craik et al., 1999). The structure and amino acid sequence of representative members from these two subfamilies are shown in Figure 1.

Despite extensive screening, until recently cyclotides had only been found in two phylogenetically distant families: the Violaceae and the Rubiaceae, with the exception of two more distantly related trypsin inhibitor peptides also carrying a CCK motif, found in the Cucurbitaceae (Hernandez et al., 2000; Felizmenio-Quimio et al., 2001; Heitz et al., 2001; Gruber et al., 2008; Koehbach et al., 2013a). However, cyclotides have now also been identified in members of the Fabaceae (Poth et al., 2010; Nguyen et al., 2011) and Solanaceae (Poth et al., 2012), and are probably also present in other families yet to be characterized.

Although the Violaceae has been identified as one of the major sources of cyclotides and cyclotide diversity, only a limited number of species have been examined. The majority of studies have been of plants of the genera *Viola* and *Hybanthus* (Broussalis et al., 2001; Simonsen et al., 2005; Ireland et al., 2006; Herrmann et al., 2008; Hashempour et al., 2013) with only a few studies having been conducted on other Violaceae genera, despite there being many of them (Hallock et al., 2000; Trabi et al., 2009; Burman et al., 2010). Consequently, sampling has been too narrow and biased for definitive conclusions to be drawn about the distribution of cyclotides among the Violaceae.

The family Violaceae comprises 23–31 genera and approximately 1050 species worldwide (Hekking, 1984, 1988; Munzinger and Ballard, 2003; Wahlert and Ballard, 2012; Wahlert et al., 2014; Tokuoka, 2008). Most genera contain a small number of species or are monotypic and restricted to the New World or Old World tropics. The three largest classical genera, *Viola, Hybanthus* sensu lato (including *Cubelium, Pigea, Pombalia*, and other proposed segregate genera), and *Rinorea* sensu lato (including *Dioryktandra* and other proposed segregated genera) account for more than 90% of Violaceae species (Tokuoka, 2008; Wahlert et al., 2014). The largest of these three genera is *Viola*, the “true violets,” which are characteristically herbs with bilaterally symmetrical, spurred flowers. The other genera have radially symmetrical flowers and are lianas, shrubs, or either large or small trees. The classification of Hekking recognizing three subfamilies are not supported by phylogenetic analyses based on nucleotide sequence data, subsuming the subfamilies Leonioideae and Violoideae into a single entity under the latter name (Tokuoka, 2008; Wahlert et al., 2014).

In the current study we undertook an extensive analysis of a broad collection of samples from within the family Violaceae. The main source of samples was from plant collections in three major Swedish herbaria. Due to the age and rarity of these samples it was necessary to make some adjustments to the current used methods to be able to identify cyclotides from the minute amounts of plant material that were available. Using this strategy we mapped the occurrence and distribution of cyclotides among the Violaceae on a large scale, analyzing more than 200 samples, representing approximately 1/6 of all species and the majority of genera. We show that certain cyclotides are more commonly expressed and occur in many species whereas other cyclotides are species specific. We conclude that cyclotides are ubiquitous in the Violaceae family of plants, that the chemical diversity of the cyclotide family is further expanded by the presence of glycosylated cyclotides, and demonstrate the exceptional stability of cyclotides by their presence as stable proteins in 200-year-old herbarium specimens.

## Results

**Figure 2:**
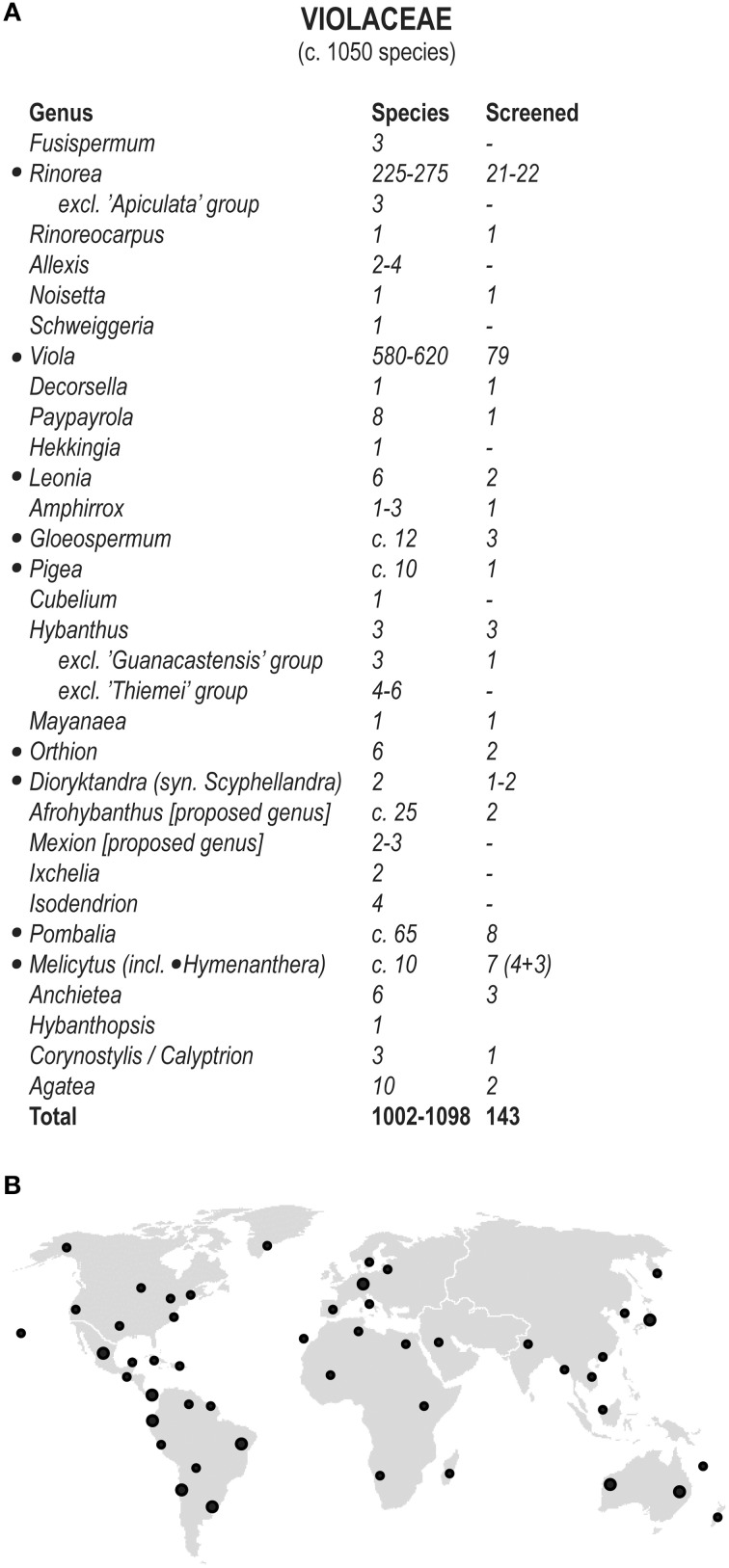
**Classification and distribution of species in ***Violaceae***. (A)** Classification of Violaceae in accordance with the phylogenetic analysis by Wahlert et al. (2014) with additional taxonomical changes (de Paula-Souza and Ballard, 2014; de Paula-Souza and Pirani, 2014; Wahlert et al., 2015). The number of recognized species positively identified to contain cyclotides is tabulated next to the total number of species. The genera containing isolated cyclotides are indicated by bullet points (•). **(B)** Geographical origins of herbarium specimens sampled for mapping cyclotide diversity and occurrence in the Violaceae. Although the family has a cosmopolitan distribution, nearly all genera are tropical. The only exceptions are species from the genus *Viola* and the species from the genus *Cubelium*.

A large-scale chemical screen was conducted on 143 species representing 17 of the possible 31 genera of Violaceae from all parts of the world. Sampling was focused in the three major classical genera, *Viola, Hybanthus* sensu lato, and *Rinorea* sensu lato, in order to include a significant sampling of all subgroups and to prevent problems that might have arisen from some genera being polyphyletic e.g., *Hybanthus* (Tokuoka, 2008; Wahlert et al., 2014). An overview of the number of samples screened from each genus, and the geographical origin of samples is shown in Figure 2. A complete list of species is found in the Supplementary Table 1.

### Screening procedure development

Plant material was sampled at three major herbaria in Sweden, namely those located in Uppsala (UPS), Gothenburg (GB), and Stockholm (S), each of which possesses extensive plant collections. We strived to ensure that the sampling covered all parts of the phylogenetic tree of Violaceae. As a large number of samples were to be analyzed, but with only limited amounts of plant material for each species, we developed a simple, fast and sensitive method for cyclotide identification based on liquid chromatography-mass spectrometry (LC-MS). Briefly, plant material was first extracted with 60% acetonitrile, the extract was diluted, and cyclotides were captured on a C18-SPE column. The eluate was freeze-dried, re-dissolved to a concentration proportional to the original amount of plant material from which the extract had been taken, and then analyzed. With this procedure it was possible to get robust LC-MS results using extracts corresponding to as little as 1 mg of plant material. However, for practical reasons, and to allow for repeated analyses, most extractions were performed on approximately 10 mg of plant material, typically corresponding to less than 1 cm^2^ of leaf material.

### Identification of cyclotides

**Figure 3:**
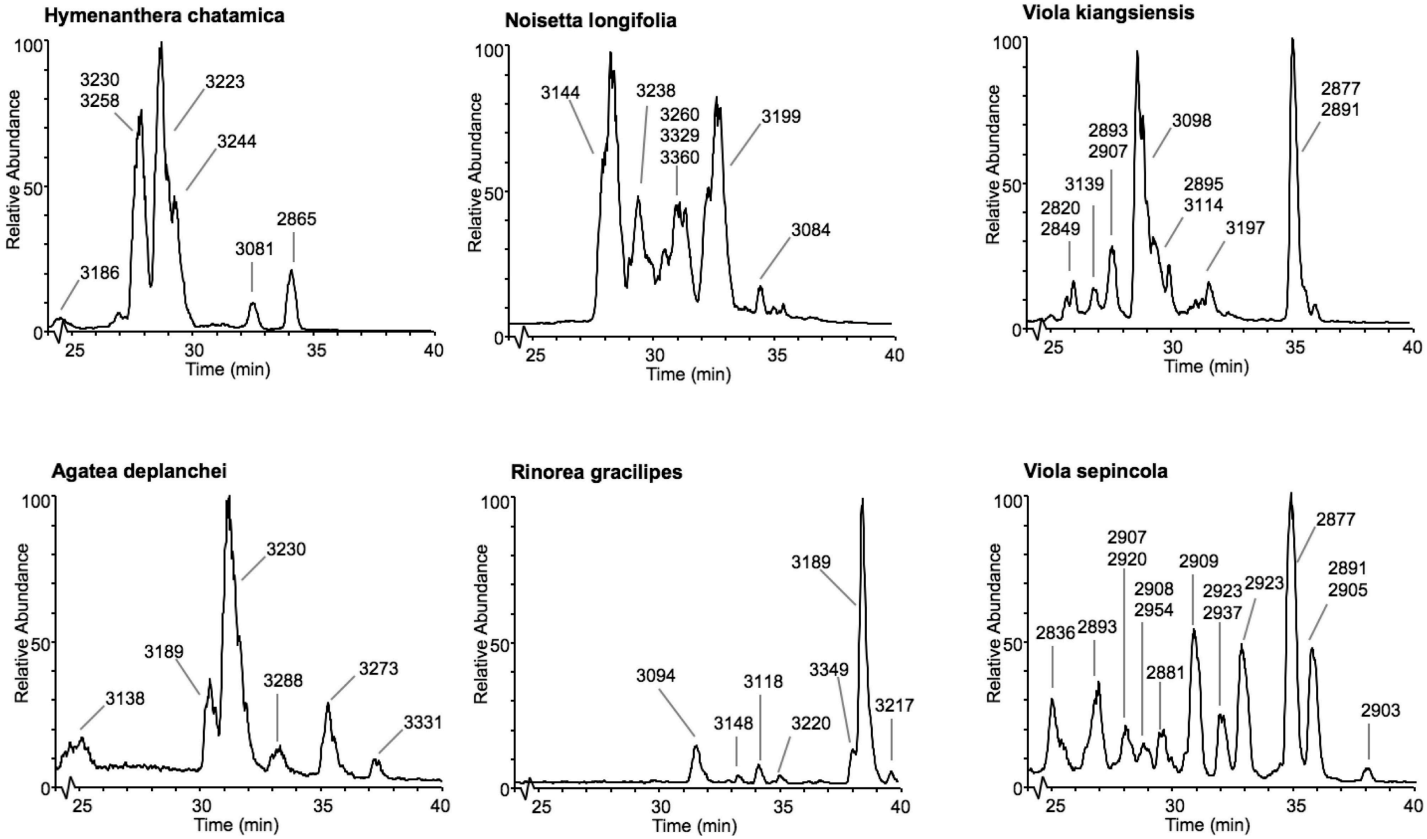
**Base peak chromatograms (m/z = 800–1900) from six representative species in the Violaceae**. The cyclotide region spanning from 25 to 40 min is shown, and major components are labeled with molecular weights (Da). Note that the LC-MS traces of *Viola kiangsiensis* and *Viola sepincola* contain more cyclotides, and also express varv A (mass 2877 eluting at 35 min), which is found in 2/3 of all *Viola* species.

**Figure 4:**
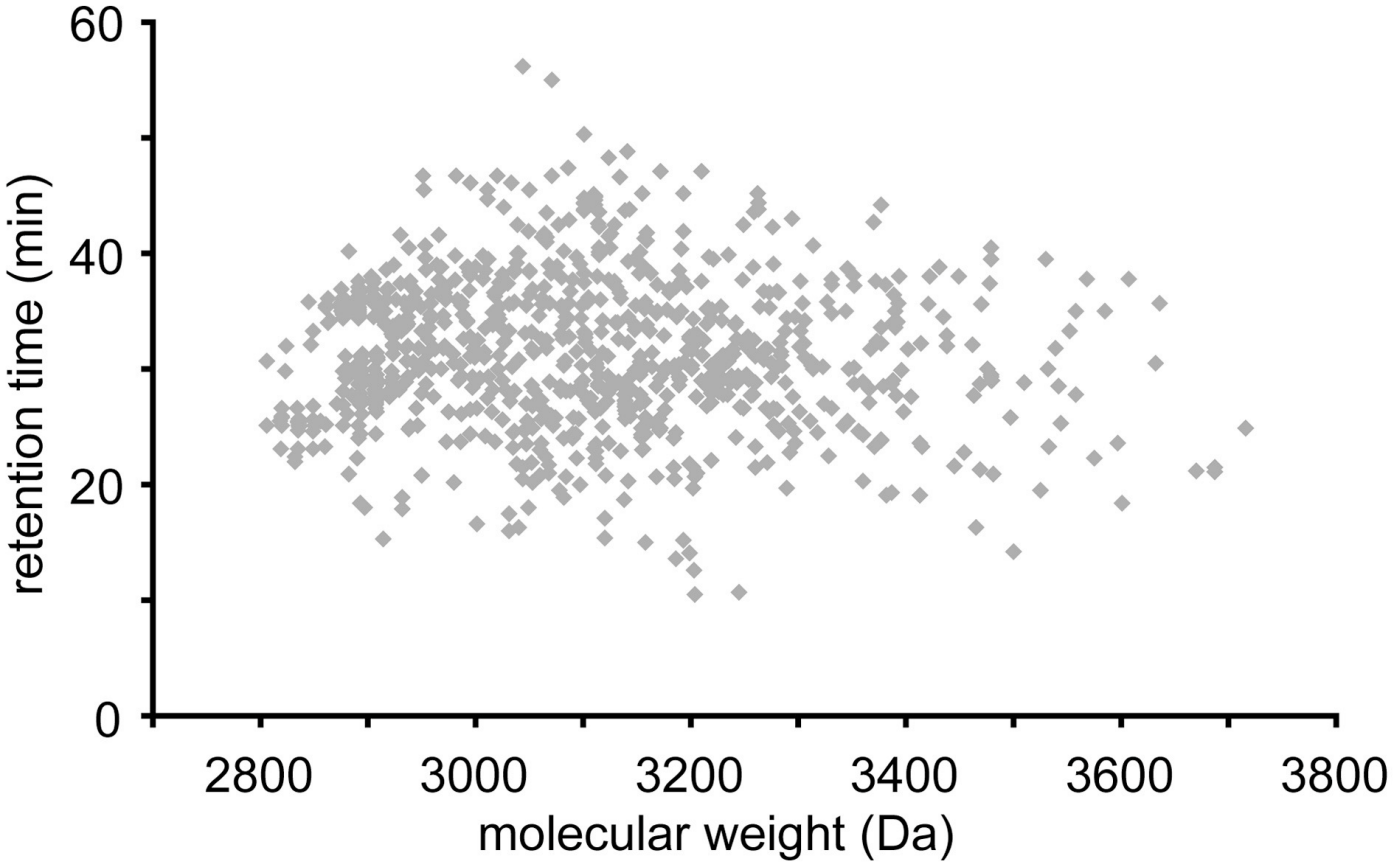
**Scatter plot data of all detected cyclotides**. Each symbol represents one of the 1043 cyclotides detected in the 143 species. Some cyclotides were found in more than one species. The vertical clusters of peaks around 2900 Da reflect the more common cyclotides such as varv A, varv F, and kalata B1. Masses range from 2800 to 3700 Da and retention times range from 15 to 60 min.

Cyclotides were putatively detected by their late retention times and molecular masses between 2.8 and 3.8 kDa. Figure 3 shows LC-MS base peak chromatograms of six species from five different genera. After visually inspecting all chromatograms, we compiled a database of the detected cyclotides. The data are given in the Supplementary Table 2 as an Excel sheet, which can be organized according to plant species, retention time (i.e., relative hydrophobicity), molecular weight, and occurrence in more than one plant species. Peaks having a unique molecular weight or retention time (±1 min) were classified as individual cyclotides. Using these criteria, 744 different cyclotides were detected from the 143 species and their retention time relative to mass is plotted in Figure 4. The masses varied from 2806 to 3716 Da with an average of 3070 Da and a median of 3048 Da.

From the analyses it is clear that the number of cyclotides varies substantially among species. In some species only a single cyclotide was detected (e.g., *Orthion oblanceolatum* and *Leonia glycocarpa*), whereas other species contained 25 or more cyclotides (e.g., *Viola cazorlensis* and *Viola allchariensis*). The number of detected cyclotides in different species did not correlate with any specific genus or with the phylogenetic pattern of the Violaceae. When the distribution of different cyclotides was compared among species, it was apparent that many cyclotides occurred in more than one species. For example, varv A was found in 70% of the analyzed species of the genus *Viola*, whereas varv E and kalata B1 were present in about 50%, and six other cyclotides were in 10–30% of the *Viola* species. The remainders of the cyclotides were only found in either one or a few (< 10%) species.

Cyclotides were positively identified in all genera tested. The number of species that was shown to contain cyclotides corresponds to 15% of the total number of species in the Violaceae family. Although some samples gave an initial negative result (less than 10%), in that no ions corresponding to cyclotides were detected using LC-MS, no correlation was seen between the distribution pattern of negative samples and genera or species. Where it was possible to resample species using samples from other sources, cyclotides were usually found. The main reason for this is most likely due to different conservation techniques that might have been used to preserve the plant material: for example, a botanist might have preserved a plant specimen whole, or sprayed it with ethanol during the preparation of the specimen in order to remove living insects, fungi and bacteria. This latter procedure might have extracted hydrophobic proteins, including cyclotides, making it difficult to detect them with our analytical protocols.

### An evolutionary perspective on the cyclotide fingerprints

The cyclotide fingerprints were then analyzed for their possible chemosystematic value, e.g., to either reinforce current knowledge on the evolution of the Violaceae or predict the evolution of cyclotide proteins *per se*. The LC-MS profiles were used for clustering analysis by UPGMA and neighbor-joining [as implemented in PAUP^*^; (Swofford, 2003)], but the lack of the same cyclotide in two or more taxa did not provide enough resolution to draw conclusions about evolution of diversity of cyclotides. An example cluster analysis is shown in the Supplementary Figure 1. To increase the data points we added complete translated prepropeptides or the corresponding mRNAs available in NCBI nucleotide (GenBank) and protein databases. This approach indicates an origin of cyclotide diversity within each taxon, but also failed to produce results in agreement with botanical classification. Thus, more information of the genetic structure of cyclotides is needed to investigate the evolution of cyclotides.

Interestingly, approximately 1/3 of the cyclotides were detected in more than one species; thus, on average approximately 2/3 of the cyclotides present in a single species are unique to that species. Hence, by exploring any uninvestigated species in the Violaceae, it should be possible to discover an entirely new set of cyclotides with novel sequences. In the present study we detected on average 5 or 6 unique sequences per species. By extrapolation, there are potentially at least 5000 different cyclotides in the Violaceae alone, which is consistent with earlier estimates (Simonsen et al., 2005). Nevertheless, this is still likely to be an underestimate of the total number of cyclotides, since we are probably only able to detect the most abundant cyclotides in this screen. Based on the experience of species investigated in detail from large scale extractions, such as *Viola odorata* (Ireland et al., 2006), the real value is probably up to five times higher, i.e., approximately 25,000.

### Sequencing of novel cyclotides

Abundant protein peaks from a diverse set of genera were purified by HPLC and reduced by dithiothreitol (DTE), followed by S-carbamidomethylation using iodoacetamide. Alkylation is an effective way to confirm the number of cysteines in a protein. Because cyclotides contain six cystines, i.e., three disulfide bonds, the reactions increase the mass by 348 Da (58 × 6 Da). The reactions were performed on almost 100 collected chromatographic peaks, all of which gave an increase of 348 Da, reinforcing their identity as cyclotides.

In order to be able to sequence small proteins efficiently with tandem MS, the proteins have to be in a linear conformation. Using microwave assisted enzymatic cleavage (together with endoproteinase GluC, which cleaves after the conserved Glu residue within cyclotides), we were able to get linear products amenable for sequencing within a matter of minutes. For full sequence coverage, the enzymatic digest was repeated with trypsin. Samples were analyzed using nanospray MS-MS using a Q-Tof MS. The procedure was performed both on known cyclotides to confirm the identity of protein peaks in the LC-MS analysis, as well as on novel cyclotides in order to identify new sequences.

In total, 12 novel cyclotides were sequenced (Table 1) from nine different species in six genera. The total number of known cyclotides is now becoming so large that a uniform naming system is necessary. The problem is likely to increase exponentially as the number of new sequences increases in the near future, not the least with the current development of total RNA sequencing. In the present study we follow the proposed naming system in which a trivial name for a cyclotide is constructed as an indicative and pronounceable acronym of the Latin binomial of the plant from which it was first isolated, followed by a letter indicating its order of discovery (Broussalis et al., 2001; Arnison et al., 2013).

**Table 1:** **Alignment of the isolated cyclotides of nine species in six different genera in the Violaceae, but the species are given as reported on herbarium sheets**.

**Species**	**Cyclotide**	Sequence^*^	**Mol weight (mono isot.)**
		**Loop 2 3 4 5 6 1**	
*Gloeospermum pauciflorum*	Glopa F	SCVFL-PC-LSVSLGCSC**K**N**K**V––CY**R**NG**R**LPCG**E**	3310.5
*Gloeospermum pauciflorum*	Glopa G	SCVFL-PC-LSAVLGCSC**K**N**K**V––CY**R**NG**R**LPCG**E**	3294.5
Hybanthus denticulatus^**^	Hyde A	SCVFD**R**TC-HLA––GCGCGSTVPLCV**R**NGVLPCG**E**	3228.4
Hymenanthera oborata^**^	Hobo A	TCTLG-TC-NTP––GCTCSW––PLCT**K**NGLPTCG**E**	2936.2
*Melicytus macrophyllus*	Mema A	SCVWL-PCTVTALLGCSC**K**D**K**V––CY**R**NGL-PCA**E**	3307.4
*Melicytus macrophyllus*	Mema B	SCVWL-PC-LTGLVGCSC**K**NNV––CYTNGTVPCG**E**	3395.3
*Orthion oblanceolatum*	Orto A	SCVYL-PCLLTAPLGCSC**K**N**K**V––CY**R**NGL-PCG**E**	3280.5
*Rinorea gracilipes*	Rigra A	SCVWL-PCTVTALLGC**K**C**E**T**R**G––CTLNGV-PCG**E**	3188.4
*Rinorea lindeniana*	Rili A	SCVWL-PCTVTALLGCTCVD**R**V––CFLDGL-PCA**E**	3262.4
*Rinorea lindeniana*	Rili B	TCAGG-TC-NTP––GCSCTW––PLCT**R**NGLPVCG**E**	2876.1
*Viola decumbens*	Vide A	SCVFL-PC-LTSALGCSC**K**S**K**V––CY**R**NGL-PCG**E**	3113.4
*Viola nivalis*	Vini A	SCVWL-PC-LSGLAGCSC**K**N**K**V––CYYDGSVPCG**E**	3216.3

*Amino acids in bold indicate where trypsin or endoproteinase GluC cleaved the proteins prior to tandem MS sequencing*.

* *As MS sequencing does not discriminate between leucine and isoleucine both are represented as L*.

** *The species Hybanthus denticulatus should otherwise be given as an as yet unnamed genus segregated from Hybanthus, and Hymenanthera obovata should be given as Melicytus obovatus*.

The novel cyclotides found in the present study resemble known sequences, but some differences are worth mentioning. For example, hyde A has an atypical sequence not characteristic of any of the two major subfamilies or hybrid variants thereof. The hybrids that have been found so far, e.g., kalata B8 (Daly et al., 2006), have a typical bracelet sequence but contain a shorter loop 3, which gives rise to an abnormal helix. Hyde A has, like kalata B8, a short loop 3 but instead of being similar to either bracelet or Möbius cyclotides in loop 5, it has a unique six amino acid sequence: GSTVPL. Most cyclotides have four amino acids in that loop, or exceptionally five amino acids, and the bracelet types often comprise 1–3 charged Lys or Arg residues, while Möbius forms have a aromatic amino acid in conjunction with a *cis*-Pro to create a conformational twist in the backbone. Hyde A also possesses a positively charged residue in loop 2, which is unusual and is seen only in a few other cyclotides.

### Glycosylation—a further increase in the diversity of cyclotides

**Figure 5:**
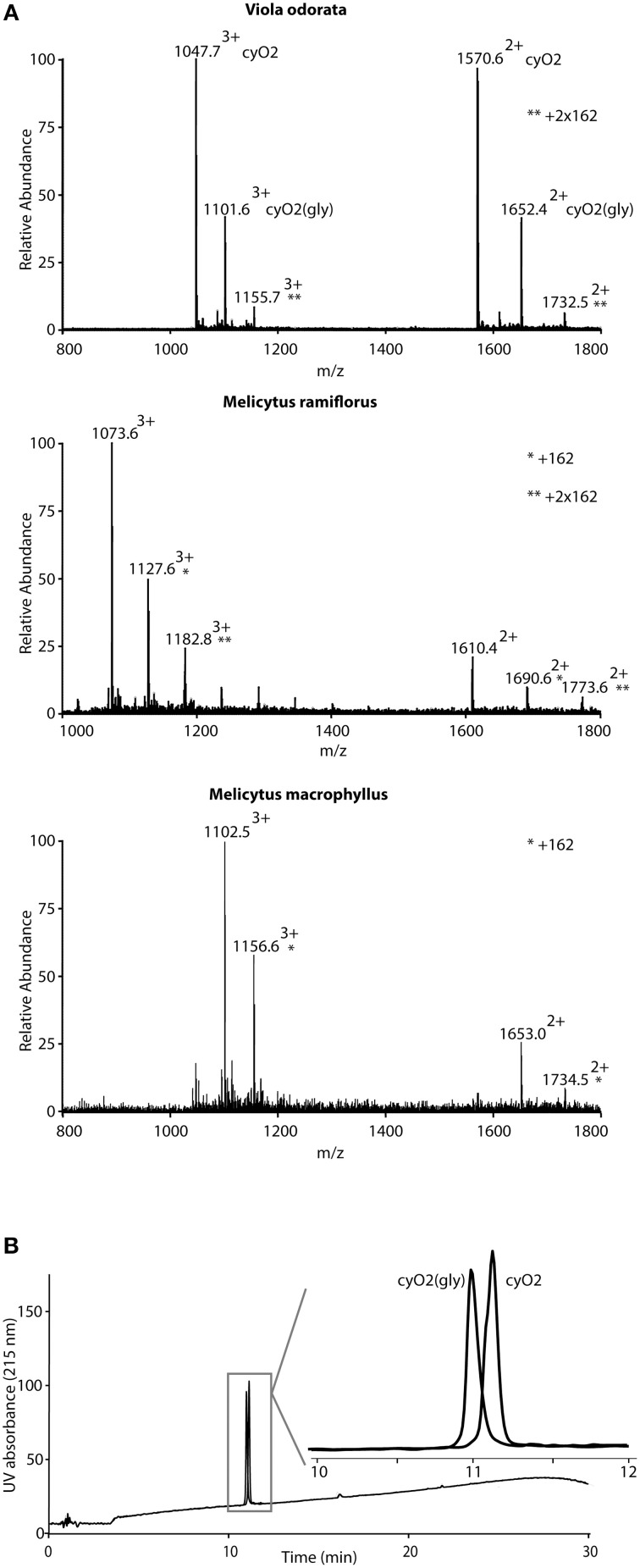
**Detection of glycosylated cyclotides**. Cyclotides with additions of 162 mass units were observed for several of the cyclotides in the screening. **(A)** Shows representative MS-data for three cyclotides and their glycosylation products from three species. At the top, *Viola odorata* containing cycloviolacin O2 (cyO2) as demonstrated by the doubly and triply charged ions (1570.6^2+^/1047.7^3+^) and the corresponding ions for cyO2(gly) (marked with ^*^). Cyclotides mram 1 from *Melicytus ramiflorus* (1610.4^2+^/1073.6^3+^) and mema A from *Melicytus macrophyllus* (1653.0^2+^/1102.5^3+^) and their respective glycosylated derivates show the same patterns of ions. Note that the cyO2 and mram 1 show signs of double glycosylations (^**^). The cyclotide derivates with additional 162 Da (corresponding to one sugar residue) shown in the figure have also been sequenced by MS-MS, demonstrating that the glycosylation is localized in loop 5. Cyclotides and their glycosylated derivates elute closely (or co-elute) on RP-HPLC, but may be separated. **(B)** Shows the separation of cyO2 and cyO2(gly), which unambiguously define them as two separate molecular species. The separation was done using a Phenomenex Kinetex C18 column (150 × 4.6 mm, 2.6 μm) operated at a flow rate of 1 ml/min and a 2%/min gradient of acetonitrile in water, containing 0.1% formic acid.

During the MS analysis it was apparent that a total of 115 cyclotides from 53 different species were observed in their expected form but also as variants carrying post translational modifications corresponding to mass additions of 162 Da or multiples of 162, consistent with one or more hexose sugars (Figure 5; cyclotides are marked in Supplementary Table 2. One of these glycosylated cyclotides was related to the well-characterized cycloviolacin O2 (cyO2) and was isolated in sufficient quantities to allow for detailed characterization. Commercial monosaccharide analysis of hydrolyzed glycosylated cyO2 [cyO2(gly)] revealed hexose to be primarily glucose, but with traces of arabinose, fucose, and galactose also being detected (data shown in Supplementary Figure 2). Extensive analysis by tandem MS spectrometry showed that the hexose moiety in glycosylated variants of cyO2, mema A, and mram 1, was associated with loop 5, i.e., KSKV or KDKV. However, definitive evidence for which residue it was attached to be difficult to obtain solely based on MS data, due to poor fragmentation in this region. Instead we used both chemical and NMR spectroscopy strategies to delineate the glycosylation site.

To investigate whether the free amine groups of Lys23 and Lys25 remained unmodified, cyO2(gly) was acetylated using acetic anhydride as previously described (Herrmann et al., 2006). After acetylation the mass of cyO2 increased with 84 Da, corresponding to the addition of two acetyl groups, suggesting that hexose was not bound to either of the lysines.

To investigate whether the addition of the hexose moiety resulted in structural changes, we structurally characterized cyO2(gly) using solution NMR. Homonuclear 2D NMR spectra were recorded, analyzed, and compared to spectra for cyO2 (Göransson et al., 2009). The NMR spectra for cyO2(gly) were of good quality in terms of signal dispersion, as generally observed for cyclotides. However, cyO2(gly) showed broader lines than native cyO2 and the spectra contained evidence of minor conformations or other inhomogeneities being present. The peaks corresponding to the main isomer were assigned using sequential assignment methods. From the secondary shifts, which are good indicators of secondary structure, it is clear that the cyclotide fold is retained after glycosylation. In particular, the Hα chemical shifts were very similar between the glycosylated and non-glycosylated forms, with the exception of the site around the modification. This was further supported by trends in the shifts of the amide protons as well as the side chain protons. Interestingly, the side chain of Lys25, which is rather flexible in solution in native cyO2, becomes more ordered in cyO2(gly), as evident from separation of the chemical shifts of the methylene pairs HD2/HD3 and HE2/HE3. However, the sugar moiety itself does not appear to be structured as no well resolved sharp peaks that could be assigned to specific protons of the sugar, or connections between the sugar and peptide, could be identified.

The combined analyses suggest that Ser24 is the site of glycosylation in loop 5 (KSKV) in cyO2(gly). Mema A do not possess an amino acid with a hydroxyl group in loop 5 (KDKV), but it may be that the hexose moiety in this case are attached to Asp25. Glycosylation, in particular O-glycosylation of Ser residues (Gomord et al., 2010), is relatively common in plants but has never been described for cyclotides. The functional role of cyO2(gly) was tested by comparing its cytotoxic activity to native cyO2 against U937-GTB lymphoma cells. Interestingly, the observed activity was identical to that for unmodified cyO2, with both having an IC_50_ of approximately 0.9 μM, and in terms of cytotoxic activity glycosylation is functionally irrelevant. Cycloviolacin O2 had an IC_50_ of 0.87 ± 0.14 μM, and the glycosylated variant 0.91 ± 0.08 μM. The dose response curve can be found in Supplementary Figure 3.

### Stability of cyclotides

**Figure 6:**
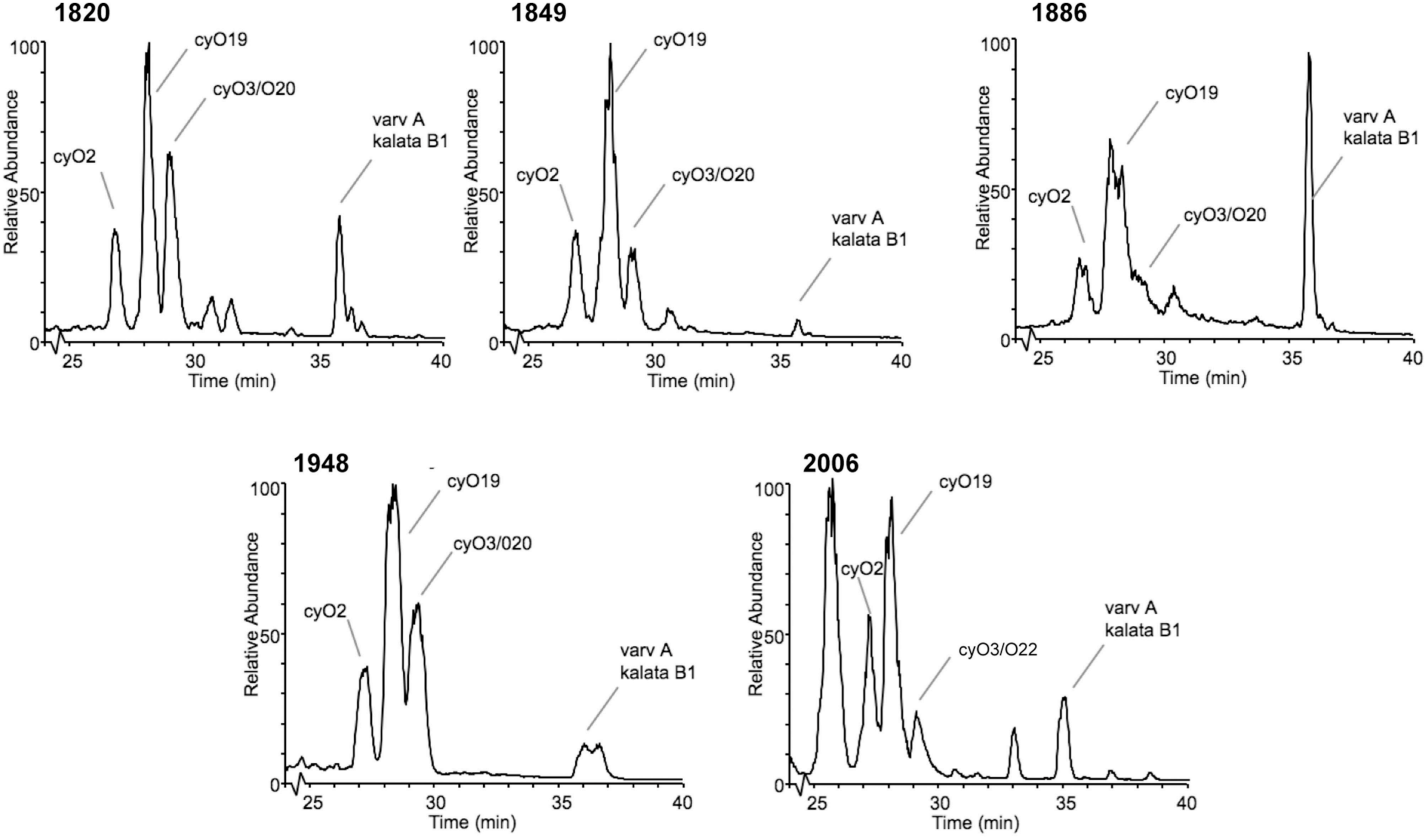
**Stability of cyclotides shown in an almost 200-year old sample**. Base peak chromatogram (m/z = 800–1900) from five specimens of *Viola odorata* collected in the years 1820, 1849, 1886, 1948, and a commercially available *Viola odorata* (2006). The cyclotide region from 25 to 40 min is shown, and the major components are labeled. Note that the major cyclotide components—the cycloviolacins O2, O19, and varv A—are present in all samples.

Access to herbaria collections made it possible to examine cyclotide stability over time periods not attainable by other means. It was also necessary to verify that cyclotides could be extracted from materials held in old plant collections. Using sweet violet, *Viola odorata*, which has a high level of cyclotide expression (Craik et al., 1999; Svangård et al., 2003; Ireland et al., 2006) and is represented in a number of different herbarium collections, we were able to sample specimens collected between 1820 and the present day. The LC-MS chromatograms of these samples and the standard reference cyclotides isolated and sequenced from recently collected and commercially available sweet violet are compared in Figure 6. The three dominant cyclotides (cycloviolacin O2, O19 and varv A) in all of the sweet violet LC-MS chromatograms had the same intensity of the peaks (equal order of magnitude) in both old and recent materials, indicating their concentration to be similar in all samples. The small differences seen are more similar to the seasonal variations reported for other cyclotides (Trabi et al., 2004). No signs of previously reported degradation products, e.g., oxidized tryptophan residues (Plan et al., 2007; Burman et al., 2011), were visible in any of the spectra, confirming the remarkable stability of cyclotides.

## Discussion

In this study we performed the first large-scale chemical screen for cyclotides in Violaceae. A key aspect of the study was the access to material from herbarium collections, which made it possible to include species from all continents and to cover a large part of the biological diversity of the Violaceae family. First, we adapted a small-scale extraction procedure applicable to the limited amount of dried plant material that we were permitted to sample from herbaria sheets. We then used the sensitivity and specificity of LC-MS to analyse the cyclotide content of the extracts. Using this strategy, 744 cyclotides were detected and compiled into a database for further analysis. Cyclotides were positively identified in 17 of the 23–31 genera, and in 15% of all the species in the Violaceae. The samples represented a wide spread of species across the Violaceae. More samples were taken from the larger genera, *Hybanthus* sensu lato, *Rinorea* sensu lato, and *Viola*, which together comprise approximately 90% of the total number of species of the family. The sampling also covered the majority of the smaller genera. The screen generated high-resolution data by mapping the cyclotide content found in species from different genera in the Violaceae, as well as in the subgroups of each genus.

Two approaches were used to confirm that MS peaks were correctly identified as cyclotides. First we confirmed by reduction and alkylation of cystines that the major peaks contained the three disulfide bonds characteristic of cyclotides. This is not full evidence that the peak corresponds to a cyclotide, but it strongly suggests so. Some peaks also correspond to already known cyclotides. Subsequently, cyclotides that were isolated in sufficient amounts were subjected to enzymatic cleavage and MS-MS sequencing. We managed to sequence 12 novel cyclotides from six of the genera (*Gloeospermum*, a *Hybanthus* segregate, *Melicytus, Orthion, Rinorea*, and *Viola*). In total, cyclotides have now been fully sequenced from seven genera in the family Violaceae [cyclotides have previously been isolated from the genus *Leonia* (Hallock et al., 2000)]. These seven genera are phylogenetically well spread out across the family (Figure 2A). From these results, we can conclude that Violaceae is an extremely rich source of cyclotides, and that cyclotides are ubiquitous throughout the family.

Although the exceptional chemical, thermal, and biological stability of cyclotides have been demonstrated in earlier studies, our approach of sampling extensively from plant material held in various herbaria gave us a unique opportunity to assess cyclotide stability over time. To this end, we sampled specimens of *Viola odorata* (sweet violet) that were collected as long ago as 1820. The base peak chromatograms from those samples were essentially identical to those from recently collected specimens, and no signs of any degradation products were visible. This result confirms the stability of cyclotides. Their compact, cyclic and knotted structure is probably the main factor accounting for this exceptional stability, as these characteristics limit opportunities for proteolysis, or for thermal or chemical degradation to occur (Colgrave and Craik, 2004).

Proteins are usually considered to be rather fragile biomolecules that are easily degraded by chemical and biotic factors. In the case of cyclotides, we show that their 3D-structures are retained intact in dry leaves for almost 200 years, and we have no doubt that they would be found intact in even older material if available. Indeed, our results establish cyclotides as one of the most stable protein families in nature. It is clear that this extended “shelf-life” of cyclotides is unparalleled in other protein families, making them ideal for applications in protein drug design.

When analysing the LC-MS data, we detected between one and 25 (average 7.3) different cyclotides per species. Detailed studies of a single species, e.g., *Viola odorata* that has previously been used as a model plant from the Violaceae, have shown it to express 30 cyclotides (Ireland et al., 2006). In comparison, the resulting numbers of the current study are relatively low; however, it should be borne in mind that we used only a very limited amount of plant material and it is likely that low abundant proteins may escape detection. The number of cyclotides from a single species in Rubiaceae is similar: *Oldenlandia affinis* has been examined exhaustively for proteins and RNA and found to contain at least 18 unique sequences (Plan et al., 2007).

Previous studies have identified deamidation and oxidation products as cyclotide derivatives that further increase the maximum number of peaks that can be detected during LC-MS (Plan et al., 2007; Burman et al., 2011). Here, we describe for the first time the presence of glycosylation of cyclotides. A glycosylated variant of cyO2 carrying a hexose moiety was purified and characterized. The hexose was located to loop 5 and chemical and structural analyses indicate it to be o-linked to Ser24. NMR studies revealed that while the overall characteristic fold of the cyO2 was retained, the glycosylation had impact on the local structure of loop 5. Although this was not found to have any significant impact on the cytotoxic activity, glycosylation altering both chemical and structural properties represents a further major increase in the diversity of naturally occurring cyclotides and is possibly functionally relevant in other biological assays. For example, outside the plant kingdom glycosylation of host defense peptides has been shown to be important for antimicrobial activity (Bulet et al., 1999).

The current study demonstrates that each species is characterized by its own set of cyclotides, many of which are unique to that species. The systematic value of the data proved to be limited. The cluster analysis is based on the qualitative profiles of cyclotides in Violaceae, but including sequence data did not improve the analysis (data not shown). Comparison of cyclotide sequences is complicated by the presence of identical amino acid sequences in distantly related species (e.g., kalata B1 in *Viola odorata* and *Oldenlandia affinis*), as well as by the presence of multiple cyclotides sequentially encoded by the same mRNA (e.g., kalata S–kalata B1–kalata S in *Viola odorata*). This leads to problems in deducing homology among the peptides, and thus phylogenetic analysis based on amino acid sequences can be distorted. Other means of increasing the number of data points are analysing the complete translated prepropeptides or the corresponding mRNAs, which we tested on sequences available in NCBI nucleotide (GenBank) and protein databases. These approaches indicate an origin of cyclotide diversity within each taxon, but also failed to produce results in agreement with botanical classification. Thus, more information of the genetic structure of cyclotides is needed to investigate the evolution of this group of proteins. Although not supported by the cluster analysis, the genus *Viola* seems to hold a common set of cyclotides that are expressed in abundance and found in the majority of the species.

It has been suggested that cyclotides are expressed when a plant is stressed by external factors, such as grazing by insects or other herbivores. For instance, the cyclotide content in plants grown under glass is lower, than in plants grown in the field (Mylne et al., 2010), and another study has demonstrated the up-regulation of cyclotide-like genes after smut infection on maize plants (Basse, 2005). Thus, the fact that each species expresses its own characteristic cocktail of cyclotides may result from its combined response to a number of different stress factors during its growth. Alternatively, the fact that cyclotides are a fast evolving group of proteins, may have enabled each species to produce its unique set of cyclotides.

**Figure 7:**
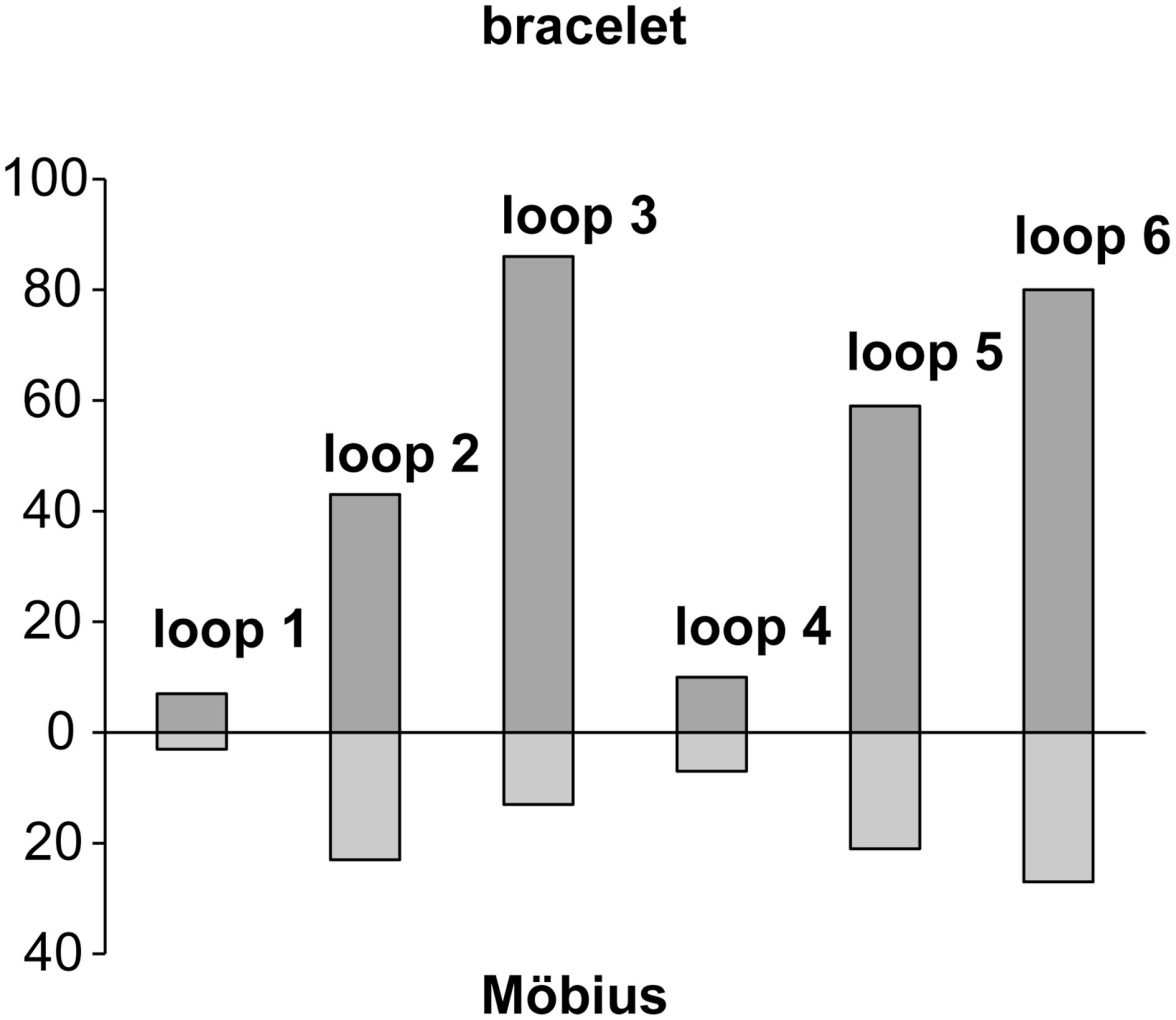
**Intercysteine loops of Möbius and bracelet cyclotides illustrating the plasticity of the cyclotide scaffold**. The total number of different loops between each of the cysteines is shown as bars in upwards direction for the bracelet (including the hybrids) and downwards for Möbius cyclotide (including linear variants). The loops with longer sequences (2, 3, 5, and 6) clearly have more variants than the loops with few amino acids (1 and 4).

To date, more than 250 cyclotide sequences have been described (see Cybase, http://www.cybase.org.au; Mulvenna et al., 2006; Wang et al., 2008b), 2/3 of which belong to the bracelet subfamily, the rest to the Möbius subfamily. In the current study we estimate the number of cyclotides in the Violaceae to be in the range of 5000–25,000, while in the Rubiaceae the number has been previously estimated to be between 10,000 and 50,000 (Gruber et al., 2008). Adding these values together gives a total of between 15,000 and 75,000, which clearly illustrates the plasticity of the cyclotide scaffold and the important role that the cyclotides must have in plant defense. It also makes the cyclotides one of the largest groups of proteins known, and taking into account the fact that they are also found in other plant families, the total number of naturally occurring cyclotides may be even higher. Figure 7 shows the total number of loops between each of the cysteines of the two main subfamilies. By simply multiplying the number of variants of each loop, a theoretical library of 600 million different cyclotides is obtained. As all of these proteins will be composed of naturally occurring intercysteine sequences, it is likely that they, if expressed or synthesized, can be folded into native like cyclotide structures. Above all, this example demonstrates the plasticity of the structure, which in combination with its ability to harness diverse bioactivities (Leta Aboye et al., 2008; Chan et al., 2011; Aboye et al., 2012; Göransson et al., 2012; Wong et al., 2012), may be used by the plant and by us for applications in biotechnology as a privileged protein scaffold.

In summary, we have mapped the occurrence and distribution of cyclotides in the Violaceae by combining sampling of materials from archived herbarium collections and modern analytical techniques. Cyclotides demonstrate remarkable stability with no detectable degradation products in samples 200 years old. Cyclotides are expressed in plants as cocktails of up to 25 different cyclotides per species. Most cyclotides found in a single species seem to be unique to that species, although some are more generally expressed among different species. In particular, the Möbius cyclotides, varv A, varv E, and kalata B1 are found in the majority of the samples in the genus *Viola*. The total number of different cyclotides in the Violaceae is at least 5000, but may be as high as 25,000. Our mapping of cyclotide containing species covered approximately 1/6 of all species in the Violaceae, and included representatives from most genera, such that we can now definitely conclude that cyclotides are ubiquitous in the Violaceae.

## Methods

### Plant material

Samples of 5–50 mg of each plant species, comprising mostly leaf material but also fruit, were obtained from the herbaria held at the Swedish Museum of Natural History, Stockholm (S), and the Universities of Gothenburg (GB), and Uppsala (UPS). A few other samples were sourced from personal collections that had voucher specimens deposited at the Uppsala University Herbarium. We included two commercially available samples, *Viola odorata* and *Viola tricolor* (Alfred Galke GmbH, Gittelde/Harz, Germany). A complete list of the analyzed samples is provided in the Supplementary Material.

### Extraction

The plant material was frozen in liquid nitrogen, ground to a powder, and extracted three times with 2.0 mL of 60% acetonitrile in 0.1% formic acid (eluent B). The extract was diluted with acidic water to 10% acetonitrile in 0.1% formic acid (eluent A), captured on a 500 mg C18 column, washed with eluent A, and eluted with eluent B. The eluate was freeze-dried and dissolved in eluent A to a concentration proportional to the original amount of extracted material (10 μL/mg).

### Liquid chromatography—mass spectrometry (LC-MS)

The extracts were analyzed by LC-MS using a Shimadzu LC10 HPLC (Shimadzu, Kyoto, Japan) connected to an LCQ electrospray ion trap MS (Thermo Finnigan, San Jose, CA), with a Grace Vydac Everest Narrowbore column (100 × 2.1 mm i.d., C18, 5 μm, 300 Å) and a linear gradient from 10 to 60% acetonitrile in 0.05% formic acid at a flow rate of 0.3 mL/min over 60 min. The capillary temperature was set at 220°C and the spray voltage at 4 kV.

### Preparative high performance liquid chromatography (HPLC)

Preparative RP-HPLC, using a Shimadzu LC10 system equipped with an SPD-M10Avp photodiode array detector, or an Äkta system (Amersham Biosciences, Uppsala, Sweden) collecting data at 215, 254, and 280 nm, was carried out using a Grace Vydac Everest (250 × 4.6 mm i.d., C18, 5 μm, 300 Å) or a Phenomenex column (250 × 4.6 mm i.d., C18, 5 μm, 300 Å), run with linear gradients from 10 to 60% acetonitrile, with 0.1% trifluoroacetic acid, in water. Fractions were analyzed using MS.

### Reduction, alkylation, and enzymatic digestion

Isolated proteins were reduced by dithiothreitol (DTE) in 0.25 M Tris-HCl containing 1.0 mM EDTA and 6 M guanidine-HCl (pH 8.5) in the dark under nitrogen for 3 h. For S-carbamidomethylation, 50 mg iodacetamide were dissolved in 0.5 M Tris-HCl, and 2 mM EDTA was added to the reduced samples. After 10 min the reactions were quenched by adding 250 μl 0.5 M citric acid. The S-carbamidomethylated proteins were purified directly and desalted directly by HPLC. Digestion was done using trypsin and/or endoproteinase GluC dissolved in 25 mM NH_4_HCO_3_ using a Discover® microwave system (CEM, Matthews, NC) at 55°C, ΔT 5°C with the power 45 W, and a run time of 15 min.

### MS sequencing

The cleaved samples were dissolved in 50% methanol with 1% formic acid and analyzed with MS-MS. The samples were injected by syringe through a PicoTip® emittor at 0.3 μL/min connected to a Q-Tof Micro™ (Waters, Milford, MA) with the voltage set at 1.4 kV. The fragmentation spectra were analyzed with the help of MassLynx/BioLynx software (MaxEnt3 and Protein Sequencing).

### Monosaccharide analysis

Glycosylated cycloviolacin O2 was sent to Glycosolutions (Worchester, MA) for monosaccharide analysis (HPAEC Neutral Monosaccharide Analysis). The monosaccharide position in three cyclotides (cycloviolacin O2, mema A, and mram 1) was analyzed using MS-MS.

### NMR spectroscopy

For NMR spectroscopy studies of cyO2(gly), 2 mg of peptide was dissolved in 0.5 ml of 90% H_2_O/10% D_2_O, pH approximately 4 and 2D homonuclear datasets, including DQF-COSY, TOCSY, and NOESY were recorded at 298 K and 600 MHz on a Bruker Avance II spectrometer. All data were recorded and processed using Topspin 3.0 (Bruker) and analyzed using CARA (Keller, 2004). Chemical shifts were compared to random coil chemical shifts described by Wishart et al. (1995).

### Cytotoxicity assay

The cytotoxicity of individual cyclotides and mixtures was determined using the fluorometric microculture cytotoxicity assay (FMCA) (Larsson and Nygren, 1989; Lindhagen et al., 2008) with the human lymphoma cell line U-937 GTB. The cytotoxic properties of dilution series of cycloviolacin O2 with and without the monosaccharide were tested in duplicates at three occasions as described before (Lindholm et al., 2002; Svangård et al., 2004; Herrmann et al., 2008).

### Conflict of interest statement

The authors declare that the research was conducted in the absence of any commercial or financial relationships that could be construed as a potential conflict of interest.

## Supplementary material

Supplementary Table 1 **Species that contain cyclotides, together with collection data**.

Supplementary Table 2 **Database of detected cyclotides**.

Supplementary Figure 1 **Tree from UPGMA clustering of presence-absence of cyclotides from the LC-MS profiles**.

Supplementary Figure 2 **Sugar analyses using HPLC and electrochemical detection demonstrating that glucose is released after chemical hydrolysis**.

Supplementary Figure 3 **Dose response curve of cyO2 and glycosylated cyO2 in the cytotoxicity assay**.
