# Supplementary material for: Distribution of circular proteins in plants: large-scale mapping of cyclotides in the Violaceae
Source: Front Plant Sci. 2015 Oct 27;6:855. doi: 10.3389/fpls.2015.00855 (PMC4621522; doi:10.3389/fpls.2015.00855)
Supplement: Supplementary Table 1 — Species that contain cyclotides, together with collection data. [file Table1.DOC]

**Supplementary Table 1.** Species that contain cyclotides, together with collection data. If the collection date/collection place was incomplete or unreadable on the herbaria sheet it is labeled as XX or –.

| **Species** | **Collection place** | **Collection date**  (YYYY-MM-DD) | **Herbarium** | **Note** |
| --- | --- | --- | --- | --- |
| *Agatea violaris* | Fuji | 1953-10-01 | Stockholm |  |
| *Agatea deplanchei* | New Caledonia | 1969-02-22 | Gothenburg |  |
| *Amphirrox longifolia* | Brazil | 1983-08-05 | Gothenburg |  |
| *Amphirrox longifolia* | Brazil | XX-02-22 | Stockholm |  |
| *Anchietea frangulifolia* | Colombia | 1942-03-25 | Stockholm |  |
| *Anchietea parvifolia* | Argentina | 1984-11-03 | Uppsala | *V-222945* |
| *Anchietea salutaris* | Argentina | 1948-10-28 | Gothenburg |  |
| *Corynostylis arborea* | Peru | 1940-11-14 | Uppsala | V-222946 |
| *Decorsella paradoxa* | West Africa | 1988-11-14 | Stockholm |  |
| *Gloeospermum blakeanum* | Panama | 2005-10-18 | Uppsala | V-195986 |
| *Gloeospermum equatoriense* | Ecuador | 1980-02-14 | Gothenburg |  |
| *Gloeospermum pauciflorum* | Panama | 2005-10-18 | Uppsala | V-195988 |
| *Hybanthus attenuatus* | Ecuador | 1977-02-11 | Gothenburg |  |
| *Hybanthus bigibbosus* | Argentina | 1989-09-22 | Gothenburg |  |
| *Hybanthus calceolaria* | Brazil | 1969-10-07 | Gothenburg |  |
| *Hybanthus chodati* | Paraguay | 1973-12-30 | Stockholm |  |
| *Hybanthus communis* | Argentina | 1946-07-02 | Stockholm |  |
| *Hybanthus densifolius* | South West Africa | 1963-05-31 | Stockholm |  |
| *Hybanthus denticulatus* | Panama | 2005-10-19 | Uppsala | V-195983 |
| *Hybanthus denticulatus* | Panama | 2005-10-18 | Uppsala | V-195985 |
| *Hybanthus enneaspermus* | South West Africa | 1963-04-24 | Stockholm |  |
| *Hybanthus floribundus* | Australia | 1990-08-16 | Stockholm |  |
| *Hybanthus havanensis* | Dominicana | 1985-10-30 | Stockholm |  |
| *Hybanthus parviflorus* | South Africa | 1973-10-30 | Stockholm |  |
| *Hybanthus prunifolius* | Panama | 2005-10-18 | Uppsala | V-195984 |
| *Hybanthus urbanianus* | Cuba | 1919-07-10 | Stockholm |  |
| *Hybanthus verticillatus* | Texas | 1924-05-10 | Gothenburg |  |
| *Hybanthus yucatanensis* | Yucatan | XX-XX-XX | Stockholm |  |
| *Hymenanthera banksii* | Australia | 1935-02-28 | Gothenburg |  |
| *Hymenanthera chathamica* | Botanical Garden | 1943-03-16 | Gothenburg |  |
| *Hymenanthera dentata* | Australia | 1995-09-08 | Stockholm |  |
| *Hymenanthera obovata* | – | 1925-02-XX | Stockholm |  |
| *Leonia cymosa* | Ecuador | 1990-02-XX | Gothenburg |  |
| *Leonia glycycarpa* | Ecuador | 1992-09-14 | Gothenburg |  |
| *Mayanaea caudata* | Guatemala | 1972-03-21 | Stockholm |  |
| *Melicytus lanceolatus* | New Zealand | 1956-04-19 | Uppsala | V-222948 |
| *Melicytus macrophyllus* | New Zealand | 1938-10-17 | Gothenburg |  |
| *Melicytus ramiflorus* | New Zealand | 1980-11-02 | Uppsala | V-222950 |
| *Noisetta longifolia* | – | 1969-01-30 | Stockholm |  |
| *Orthion oblanceolatum* | Mexico | 1964-02-08 | Stockholm |  |
| *Orthion subsessile* | Guatemala | 1971-05-28 | Stockholm |  |
| *Paypayrola grandiflora* | Brazil | 1978-11-13 | Uppsala | V-222949 |
| *Rinorea anguifera* | Malaysia | 1992-03-15 | Stockholm |  |
| *Rinorea angustifolia* | Madagascar | 1999-11-28 | Stockholm |  |
| *Rinorea australasica* | Australia | 1929-11-29 | Stockholm |  |
| *Rinorea belizensis* | Guatemala | 1972-03-XX | Stockholm |  |
| *Rinorea convallariiflora* | Kenya | 1957-08-04 | Uppsala | V-222951 |
| *Rinorea convallarioides* | Southern Rhodesia | 1969-09-13 | Stockholm |  |
| *Rinorea dasyadena* | Panama | 2005-10-19 | Uppsala | V-195992 |
| *Rinorea elliptica* | Tanzania/Kenya | 1971-07-17 | Stockholm |  |
| *Rinorea flavescens* | Ecuador | 1980-03-20 | Gothenburg |  |
| *Rinorea gracilipes* | Burundi | 1981-03-11 | Stockholm |  |
| *Rinorea greveana* | Madagascar | 1931-09-30 | Gothenburg |  |
| *Rinorea guianensis* | Brazil | 1977-04-21 | Stockholm |  |
| *Rinorea hummelii* | Mexico | 1987-10-05 | Stockholm |  |
| *Rinorea ilicifolia* | South Africa | 2003-04-10 | Stockholm |  |
| *Rinorea lindeniana* | Panama | 2005-10-30 | Uppsala | V-195994 |
| *Rinorea microglossa* | Cameroon | 1970-04-01 | Uppsala | V-222952 |
| *Rinorea passoura* | Panama | 2005-10-19 | Uppsala | V-195990 |
| *Rinorea sclerocarpa* | – | – | Stockholm |  |
| *Rinorea squamata* | Panama | 2005-10-19 | Uppsala | V-195991 |
| *Rinorea subintegrifolia* | Cameroon | 1913-XX-XX | Stockholm |  |
| *Rinorea virgata* | Laotica | 1996-06-17 | Stockholm |  |
| *Rinorea viridifolia* | Ecuador | 1990-05-XX | Gothenburg |  |
| *Rinorea welwitschii* | Congo | 1951-11-XX | Uppsala | V-222954 |
| *Rinoreocarpus ulei* | Brazil | 1933-07-15 | Stockholm |  |
| *Viola adunca* | Canada | 1955-09-29 | Gothenburg |  |
| *Viola allchariensis* | Macedonia | 1921-08-07 | Stockholm |  |
| *Viola avatschensis* | Kamtchatka | 1922-07-11 | Stockholm |  |
| *Viola bertolonii* | – | – | Stockholm |  |
| *Viola biflora* | Sweden | – |  | private coll. |
| *Viola blanda* | America Orientalis | 1931-05-15 | Stockholm |  |
| *Viola brevistipulata* | – | 1961-06-06 | Stockholm |  |
| *Viola canadensis* | Canada | 1969-06-04 | Uppsala | V-222956 |
| *Viola canescens* | Himalaya | 1935-07-21 | Stockholm |  |
| *Viola canina x riviniana* | Sweden | 1953-05-28 | Uppsala | V-222588 |
| *Viola cazorlensis* | Spain | – | Gothenburg |  |
| *Viola cazorlensis* | Spain | 1904-XX-XX | Stockholm |  |
| *Viola cerasifolia* | Brazil | 1968-11-11 | Stockholm |  |
| *Viola chaerophylloides* | Japan | 1959-04-16 | Stockholm |  |
| *Viola cheiranthifolia* | Canary Islands | 1954-06-22 | Gothenburg |  |
| *Viola cinerea* | Egypt | 1997-04-13 |  | private coll. |
| *Viola collina* | – | 1956-05-04 | Stockholm |  |
| *Viola cotyledon* | Chile | 1967-12-20 | Stockholm |  |
| *Viola cucullata* | Canada | 2002-05-29 | Stockholm |  |
| *Viola dacica* | Bulgaria | 1932-07-XX | Gothenburg |  |
| *Viola decumbens* | – | 1978-06-25 | Stockholm |  |
| *Viola demetria* | Spain | 1971-06-13 | Gothenburg |  |
| *Viola dombeyana* | Ecuador | 1988-10-22 | Gothenburg |  |
| *Viola eizanensis* | Japan | 1932-07-XX | Stockholm |  |
| *Viola eminii* | Central East Africa | 1953-06-02 | Stockholm |  |
| *Viola floridana* | Mississippi | 1965-03-29 | Stockholm |  |
| *Viola formosana* | Taiwan | 1994-03-31 | Stockholm |  |
| *Viola glabella* | Canada | 1934-04-XX | Gothenburg |  |
| *Viola heterophylla* | Italy | 1961-05-17 | Stockholm |  |
| *Viola hirta* | Bayern | 1958-04-21 | Stockholm |  |
| *Viola hirtipes* | Japan | 1955-05-28 | Stockholm |  |
| *Viola kiangsiensis* | China | 1935-XX-XX | Stockholm |  |
| *Viola kitaibeliana* | Spain | 1972-04-23 | Stockholm |  |
| *Viola labradorica* | Greenland | 1976-07-24 | Stockholm |  |
| *Viola langsdorffii* | Alaska | 1977-06-17 | Stockholm |  |
| *Viola lobata* | California | 1956-06-20 | Stockholm |  |
| *Viola macedonica* | Greece | 1926-06-02 | Gothenburg |  |
| *Viola maculata* | Chile | 1940-11-18 | Stockholm |  |
| *Viola maculata* | Argentina | 1967-12-08 | Gothenburg |  |
| *Viola methodiana* | Greece | 1932-05-XX | Uppsala | V-222957 |
| *Viola minuta* | – | 1970-XX-18 | Stockholm |  |
| *Viola missouriensis* | Texas | 1946-03-23 | Stockholm |  |
| *Viola munbyana* | Algeria | 1979-05-22 | Gothenburg |  |
| *Viola nivalis* | Ecuador | 1987-12-29 | Gothenburg |  |
| *Viola nummularifolia* | Italia | 1931-07-27 | Stockholm |  |
| *Viola nuttallii* | Idaho | 1938-05-12 | Uppsala | V-222958 |
| *Viola obtusa* | Japan | 1953-05-XX | Gothenburg |  |
| *Viola ocellata* | California | 1945-10-21 | Gothenburg |  |
| *Viola odorata* | bought (GALKE) | – | – | Commercial |
| *Viola odorata* | Sweden | 1820-XX-XX | Uppsala | V-222593 |
| *Viola odorata* | Sweden | 1849-04-30 | Uppsala | V-222592 |
| *Viola odorata* | Sweden | 1886-04-20 | Uppsala | *V-222591* |
| *Viola odorata* | Sweden | 1948-05-04 | Uppsala | V-222589 |
| *Viola odorata x hirta* | Sweden | 1946-04-21 | Uppsala | V-186071 |
| *Viola pallens* | Minnesota | 1958-05-25 | Stockholm |  |
| *Viola palustris* | Germany | 1979-05-24 | Gothenburg |  |
| *Viola parnassifolia* | California | 1968-06-11 | Stockholm |  |
| *Viola parvifolia* | Ecuador | 1988-03-05 | Gothenburg |  |
| *Viola pedata* | Texas | 1947-04-04 | Stockholm |  |
| *Viola pedunculata* | California | 1965-03-25 | Gothenburg |  |
| *Viola principis* | China | 1996-04-11 | Uppsala | V-158520 |
| *Viola pubescens* | Canada | 2005-06-03 | Stockholm |  |
| *Viola pygmaea* | Bolivia | 1981-02-19 | Stockholm |  |
| *Viola rafinesquii* | Ohio | 1990-05-09 | Gothenburg |  |
| *Viola renifolia* | Canada | 1935-08-20 | Stockholm |  |
| *Viola robusta* | Hawaii | 1938-07-06 | Stockholm |  |
| *Viola rossii* | Korea | 1961-XX-XX | Stockholm |  |
| *Viola rostrata* | Japan | 1961-05-30 | Stockholm |  |
| *Viola rupestris* | Switzerland | 1932-05-08 | Gothenburg |  |
| *Viola scandens* | Ecuador | 1980-11-06 | Gothenburg |  |
| *Viola scorpiuroides* | Egypt | 2004-02-04 |  | private coll. |
| *Viola scotophylla* | Italy | 1952-04-05 | Stockholm |  |
| *Viola selkirkii* | Japan | 1955-06-26 | Stockholm |  |
| *Viola septentrionalis* | Canada | 1954-05-29 | Gothenburg |  |
| *Viola sepincola* | – | – | Gothenburg |  |
| *Viola sieberiana* | Australia | 1985-11-08 | Gothenburg |  |
| *Viola somalensis* | Somalia | 1982-02-09 | Uppsala | V-222959 |
| *Viola steinbachii* | – | – | Stockholm |  |
| *Viola stipularis* | Panama | – | – | private coll. |
| *Viola striata* | Pennsylvania | 1970-05-09 | Stockholm |  |
| *Viola suavis* | – | 1974-05-21 | Gothenburg |  |
| *Viola sumatrana* | Malaysia | 1992-03-21 | Stockholm |  |
| *Viola tracheliifolia* | – | 1912-07-XX | Gothenburg |  |
| *Viola tricolor* | bought (GALKE) | – | – | Commercial |
| *Viola turkestanica* | Middle East | – | Stockholm |  |
| *Viola verecunda* | – | 1980-04-26 | Gothenburg |  |
| *Viola yedoensis* | – | – | – | private coll. |
| *Viola zoysii* | Italy | 1934-05-10 | Gothenburg |  |
