# Supplementary figures and images for: Distribution of circular proteins in plants: large-scale mapping of cyclotides in the Violaceae
Source: Front Plant Sci. 2015 Oct 27;6:855. doi: 10.3389/fpls.2015.00855 (PMC4621522; doi:10.3389/fpls.2015.00855)

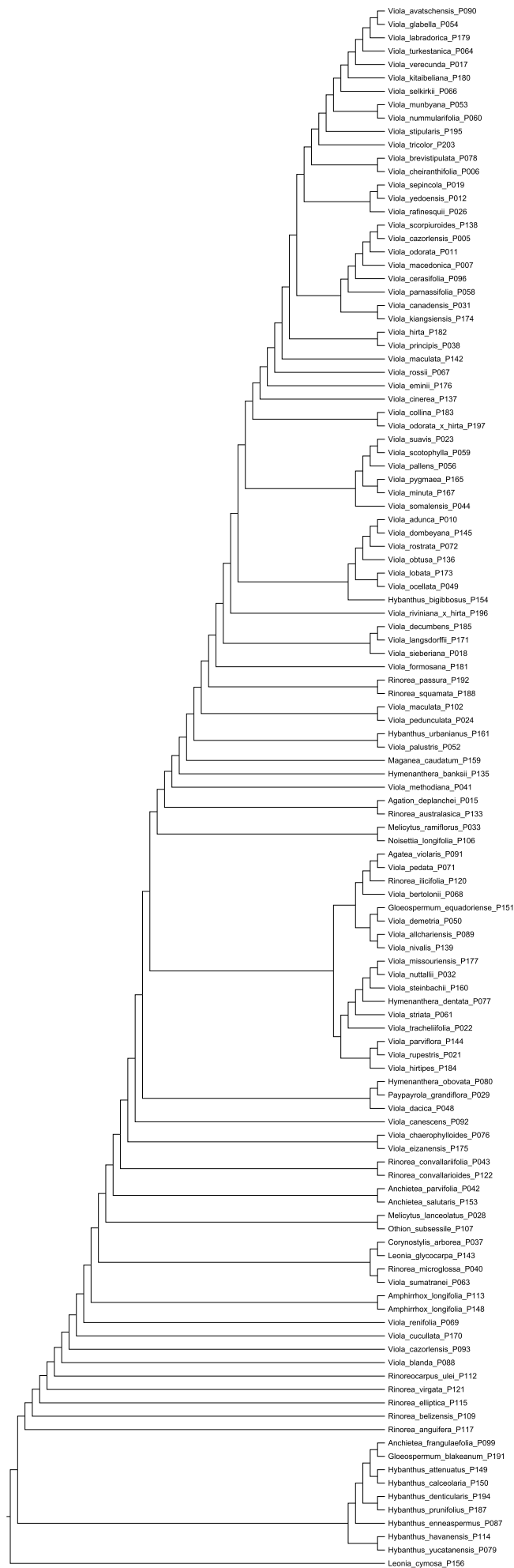

Supplement: Supplementary Figure 1 — Tree from UPGMA clustering of presence-absence of cyclotides from the LC-MS profiles. [file Image1.PDF]
