# Supplementary material for: Distribution of circular proteins in plants: large-scale mapping of cyclotides in the Violaceae
Source: Front Plant Sci. 2015 Oct 27;6:855. doi: 10.3389/fpls.2015.00855 (PMC4621522; doi:10.3389/fpls.2015.00855)

CyO2-Glyco

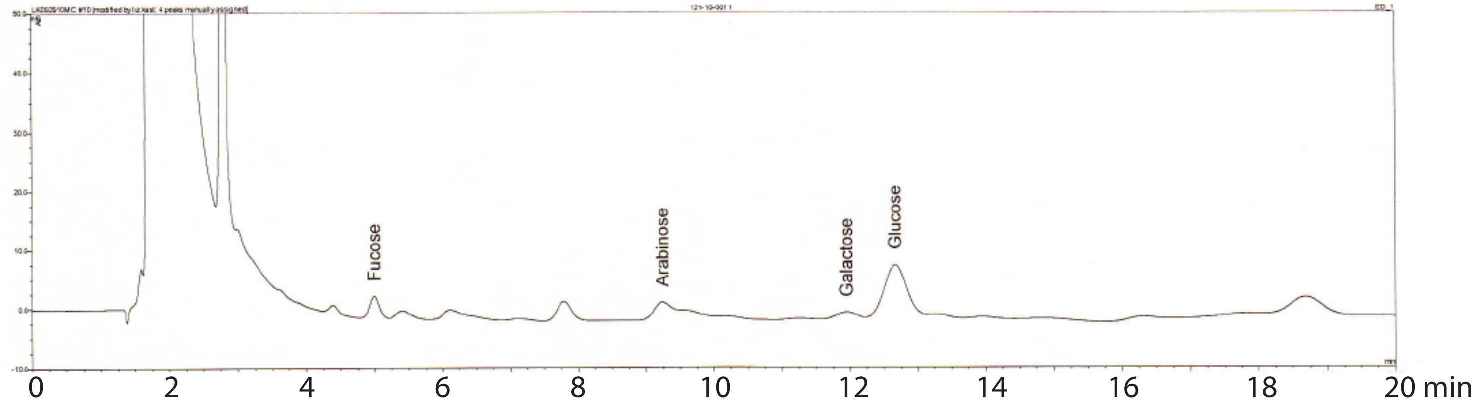

Standard Curve (Fucose, GalNAc, GlcNAc, Gal, Man)

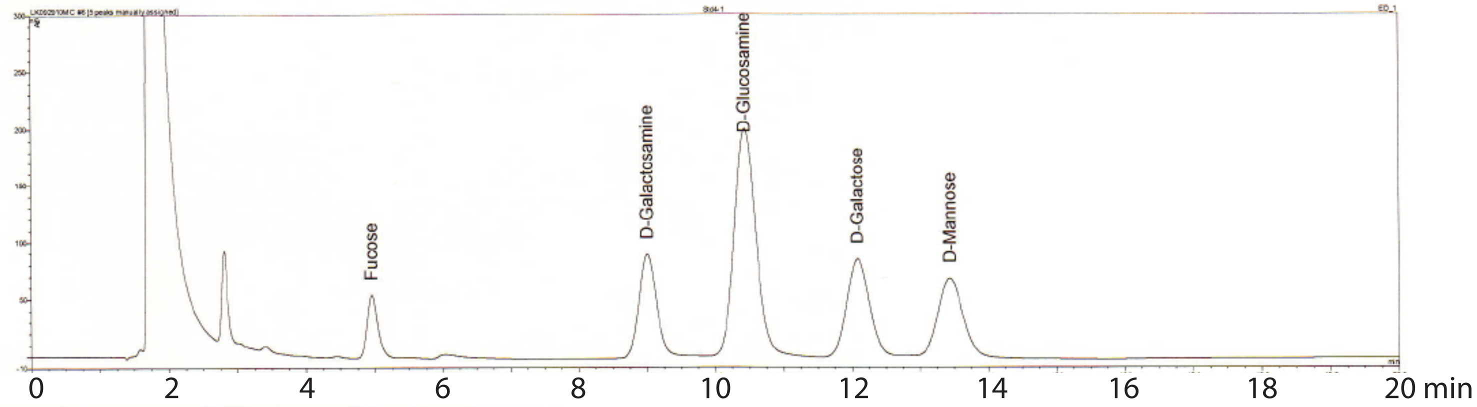

Arabinose and Glucose Standard

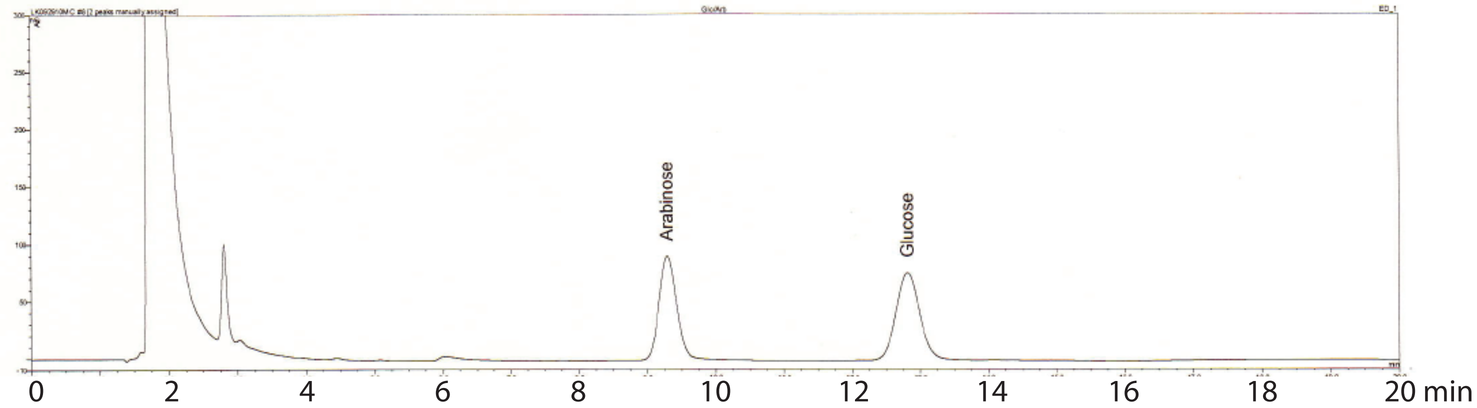

Acid Hydrolysis Control

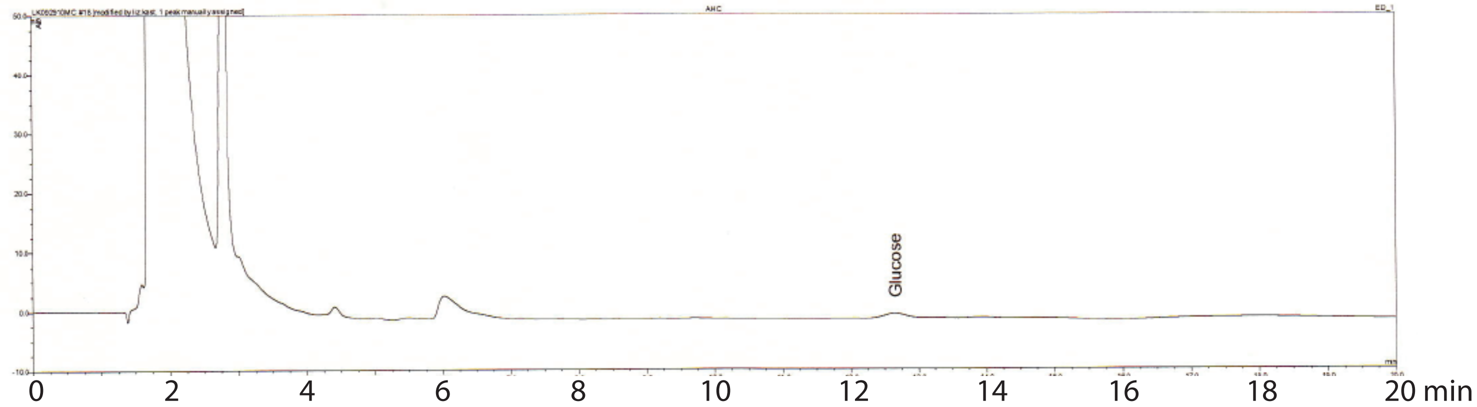

Supplement: Supplementary Figure 2 — Sugar analyses using HPLC and electrochemical detection demonstrating that glucose is released after chemical hydrolysis. [file Image2.PDF]
