# Supplementary material for: Distribution of circular proteins in plants: large-scale mapping of cyclotides in the Violaceae
Source: Front Plant Sci. 2015 Oct 27;6:855. doi: 10.3389/fpls.2015.00855 (PMC4621522; doi:10.3389/fpls.2015.00855)

Supplementary Figure 3. Dose response curve in the flurometric microculture cytotoxicity assay. Cycloviolacin O2 had an IC_{50} of 0.87±0.14 μM, and the glycosylated variant 0.91±0.08 μM.

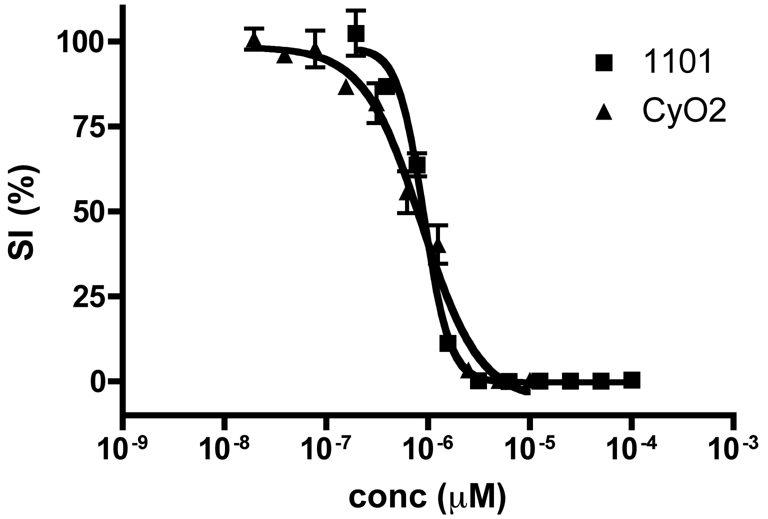

Supplement: Supplementary Figure 3 — Dose response curve of cyO2 and glycosylated cyO2 in the cytotoxicity assay. [file Image3.PDF]
